# Precision Cancer Diagnostics: Tracking Genomic Evolution in Clinical Trials

**DOI:** 10.1371/journal.pmed.1002177

**Published:** 2016-12-06

**Authors:** Francisco Beca, Andrew H. Beck

**Affiliations:** 1 Department of Pathology and Cancer Research Institute, Beth Israel Deaconess Cancer Center, Boston, Massachusetts, United States of America; 2 Harvard Medical School, Boston, Massachusetts, United States of America

## Abstract

In a Perspective, Francisco Beca and Andrew Beck discuss Charles Swanton and colleagues' accompanying Research Article on somatic mutations in patients with inflammatory breast cancer treated in a Phase II clinical trial.

It has been more than 15 years since *Time* magazine published a cover story highlighting the success of the targeted drug imatinib in the fight against chronic myeloid leukemia [1]. At the time, we were still two years away from completion of the first human genome sequence as part of the Human Genome Project, and the cost of sequencing an entire human genome was estimated to be around US$100 million [2]. Fifteen years later, sequencing technologies have evolved tremendously and have driven down the cost of sequencing ~100,000-fold. Today, genomic profiling of patients’ tumors using next-generation sequencing is readily available from a variety of academic institutions and commercial vendors. The idea of using genomic sequencing data in cancer diagnostics is now more appealing than ever, and there is great hope in the community that the increasing availability of genomic data will result in more success stories for precision cancer medicine, in which knowledge of the genetic mechanism of disease will lead to development of more active therapies and improved patient outcomes.

Breast cancer has been a major focus of research efforts in precision oncology. For decades, treatment allocation to hormonal therapy has been based on biomarker profiles. Breast cancer is one of the first solid malignancies in which a defined genomic event with clear clinical and pathological implications, human epidermal growth factor receptor 2 (HER2) amplification, was found and for which a targeted agent that clearly modifies the natural history of the disease was made readily available [3]. Due in part to these successes, breast cancer survivorship is presently measured in intervals of five or more years, rather than in single years or months. But that is not the case for all breast cancer types. Inflammatory breast cancer (IBC) is a particularly aggressive form of breast cancer that tends to affect younger women. IBC is frequently HER2-overexpressing, and the disease’s hallmark is the clinical inflammatory symptoms on the breast skin due to numerous dermal lymphatic emboli. Like in other cancer types, genomic profiling has led to identification of multiple genes that are involved in IBC [4,5]. Assessments of molecular features and quantitative measures of intratumor heterogeneity are now in the translation phase from the research setting, and their clinical validity is being tested [6,7].

In the accompanying research article now published in *PLOS Medicine*, Charles Swanton and coworkers report the results of an open-label, Phase II trial of afatinib, a tyrosine kinase inhibitor selective for ErbB family receptors, for the treatment of HER2-positive IBC [8]. This is one of the first clinical trials to prospectively integrate longitudinal whole exome sequencing (WES) in a trial for drug development and to our knowledge the first reported in IBC. Swanton and colleagues’ trial had a small final sample size and shortened trial duration because of the reporting of the results of the LUX-Breast 1 trial, in which an afatinib-containing regimen was associated with shorter overall survival and was less tolerable than a regimen containing trastuzumab in HER2-positive metastatic breast cancer. But, despite this, the investigators were able to show clinical responses with afatinib, with or without vinorelbine, in trastuzumab-naïve HER-2 positive IBC. Additionally, in nine patients it was possible to compare the clonal architecture of the tumors before and after treatment because of the planned exome sequencing at two distinct time points. In the majority of patients for whom it was possible to compare the clonal architecture of the tumors before and after treatment, the clonal composition remained largely the same between the two time points, without definitive evidence of an evolutionary bottleneck. As the authors posit, several factors could explain this. Perhaps the simplest explanation is that, since in eight of these patients there was no clinical benefit from therapy, there may not have been enough selective pressure to drive tumor evolution—in contrast to the mechanism by which *ESR1* mutations emerge in breast cancer during the course of endocrine treatment [9].

Although most patients in Swanton and colleagues’ study did not show definitive evidence of clonal evolution or benefit from therapy, there was a significant clonal shift in one of the patients. This important observation was only possible because of the prospectively planned WES analysis, demonstrating the ability of prospectively planned genomic profiling within the setting of a clinical trial to improve knowledge about IBC in individual patients, even when the study’s overall sample size is small and the overall therapeutic benefit of the experimental treatment is not established. We expect that future clinical trials in oncology will increasingly incorporate prospective deep genomic tumor characterization into their study designs, resulting in new biological insights for individual cancer patients regarding the causes of their disease and its progression over time.

For a disease as complex as inflammatory breast cancer, a success story resembling imatinib for chronic myelogenous leukemia is not likely to be realized; we may never identify a critical, single genomic driver and therapeutic vulnerability that is common across most IBC patients. However, it is likely that with increasing sequencing of individual tumors within the course of treatment, we will gain a deeper understanding of how IBC responds and progresses in individual patients, which may lead to better and safer therapies tailored to the dynamic genomic evolution of an individual patient’s disease.

## Abbreviations

HER2: human epidermal growth factor receptor 2
IBC: inflammatory breast cancer
WES: whole exome sequencing
